# Preparation of Nano-Copper Sulfide and Its Adsorption Properties for 17α-Ethynyl Estradiol

**DOI:** 10.1186/s11671-020-3274-6

**Published:** 2020-02-22

**Authors:** Sifeng Zhang, Wenxiang Meng, Lulu Wang, Lingxin Li, Yanju Long, Yunrui Hei, Luting Zhou, Shenglan Wu, Ziguang Zheng, Lijun Luo, Fengzhi Jiang

**Affiliations:** 1 School of Chemical Science and Technology, Yunnan University, Kunming, 650091 China; 2 School of Chemistry and Environment, Yunnan Minzu University, Kunming, 650500 China

**Keywords:** Copper sulfide, Adsorption, 17α-Ethynyl estradiol

## Abstract

In the present work, a tubular nano-copper sulfide was successfully synthesized by hydrothermal method. The physical and chemical properties of the prepared materials were characterized by XRD, SEM, TEM, and BET. The synthesized copper sulfide was used as an adsorbent for removing 17α-ethynyl estradiol (EE2) and exhibited excellent adsorption properties. At 25 °C, 15 mg of adsorbent was applied for 50 mL of 5 mg/L EE2 solution, adsorption equilibrium was reached after 180 min, and the adsorption rate reached nearly 90%. In addition, the kinetics, isothermal adsorption, and thermodynamics of the adsorption process were discussed on the basis of theoretical calculations and experimental results. The theoretical maximum adsorption capacity of copper sulfide was calculated to be 147.06 mg/g. The results of this study indicated that copper sulfide was a stable and efficient adsorbent with promising practical applications.

## Introduction

In recent years, with the continuous development and growth of the social industries, human activities have caused serious pollution to the environment, and global environmental problems have become increasingly serious. Among these, environmental endocrine disruptors (EDCs), as mostly persistent organic pollutants (POPs), are bioaccumulative, highly toxic, have low concentration, and latent. They can get into the human body directly or indirectly through the food chain and were enriched and amplified in the living organism [1, 2]. As a result, research on the governance of EDCs has become a widespread concern in the environmental field. Among the many endocrine disruptors, estrogen and bisphenol compounds are widely used in life, among which 17α-ethynyl estradiol (EE2) is a typical one. EE2 is commonly used in contraceptives and hormone replacement therapy. However, studies have shown that EE2 can bring serious harm to living things and humans and cause diseases such as reproductive system disorders, infertility, and cancer [3–7].. Therefore, how to effectively and inexpensively remove EE2 from water is particularly urgent.

At present, there are numerous methods for removing EE2, such as physical methods (adsorption and membrane separation), biodegradation methods, and chemical methods (oxidation methods and photocatalysis methods) [8–11]. Among these methods, the adsorption method has a promising application because of its low cost, simplicity, and no secondary pollution. So far, researchers have used biocharcoal, activated carbon, carbon nanotubes, graphene, and clay to adsorb EE2 [12–14], but the overall adsorption effect is poor and time-consuming. Yoon et al. used activated carbon to adsorb EE2 with a concentration of 100 nmol/L in water; when the dosage of activated carbon was 9 mg/L, it took 24 h to adsorb EE2 completely [8].

Copper sulfide is an important transition metal sulfide, which is extremely difficult to dissolve in water and one of the most insoluble materials [15, 16]. Nano-copper sulfide is widely used as photoconductive materials because of its low cost, simple steps, easy control of morphology, small particle size, large specific surface area, and high photothermal conversion rate. It has also potential applications in photocatalysts, thermocouples, filters, solar cells, and biomedicine [17]. In alkaline environment, the isoelectric point (IEP) of copper sulfide is large, and its surface is easy to be positive charge [18–20], while there is a phenolic hydroxyl group in the structure of EE2 [21], which can exhibit weak acidity in aqueous solution and negative surface charge, which makes strong chemisorption between them. Therefore, it is possible for copper sulfide to adsorb EE2.

In this study, a tubular nano-copper sulfide was synthesized by hydrothermal method. The specific surface area of the synthesized nano-copper sulfide was 16.94 m^2^/g, and the maximum adsorption capacity of EE2 was 147.06 mg/g. The crystal phase composition, morphology, and specific surface area of the prepared nano-copper sulfide were studied in detail. The adsorption properties of copper sulfide on EE2 were studied by optimizing the pH of the solution, the amount of adsorbent, the adsorption time, the adsorption temperature, and the initial concentration of EE2. And the kinetic adsorption, isothermal adsorption, and thermodynamic adsorption of copper sulfide on EE2 were studied through the experimental data.

## Materials and Methods

### Synthesis of the Copper Sulfide Adsorbent

All chemical reagents were of analytical grade and used without further purification. The tubular nano-copper sulfide was synthesized by hydrothermal method. In a typical procedure, 4.8 mmol of CuCl_2_·2H_2_O and 4.8 mmol of CH_3_CSNH_2_ was dissolved in 40 mL of deionized water and magnetically stirred until a clear solution was formed. Then, 20 mL of a 0.4-mol/L NaOH aqueous solution was slowly added to the above solution. After stirring for 5 min, the mixture solution was transferred to a 100-mL polytetrafluoroethylene-lined stainless steel autoclave and then was heated at 160 °C for 6 h. Subsequently, the autoclave was naturally cooled to room temperature. Finally, the solid product was centrifuged and washed alternately with ethanol and deionized water for three times and then dried at 60 °C for 6 h to obtain the material.

### Characterization

The crystal structure of the material was characterized by X-ray diffraction (XRD) using a TTRIII X-ray diffractometer (Rigaku, Japan) with CuKα radiation at 40 kV and 200 mA. The morphology of the material was investigated by a QUANTA 200 scanning electron microscope (SEM, FEI, USA) at about 20 kV and Tecnai-G20 transmission electron microscope (TEM, FEI, USA). The surface area of the nano-copper sulfide was obtained using the Brunauer-Emmett-Teller plot of N_2_ adsorption isotherm.

### Adsorption Measurements

#### Adsorption Experiment

Certain amount of adsorbent was added into the iodometric bottle containing 50.00 mL of a certain concentration of EE2 solution. Then, the iodometric bottle was put into a shaker. At a certain temperature and shaking speed of 200 rpm/min, the mixed solution was shaken for a certain time. Then, the solution was quickly filtered by a 0.4-um mixed cellulose filter to determine the concentration of residual EE2 in the solution.

EE2 concentration was detected by ultra-high performance liquid chromatography (UPLC, Waters, USA) at the detection wavelength of 210 nm. A C18 column (1.7 μm, 2.1 × 50 mm) has been employed with acetonitrile/water (55/45 v/v) at 0.35 mL/min and injection volume of 7 μL.

#### Adsorption Model

##### Adsorption Efficiency

Adsorption efficiency indicates the removal rate of EE2 by the adsorbent. The expression is as follows:

1 $$ \mathrm{Absorption}\left(\%\right)=\frac{C_0-{C}_e}{C_0}\times 100\% $$

*C*_0_ and *C*_*e*_ represent the initial concentration of EE2 (mg/L) and the concentration at which adsorption equilibrium is reached (mg/L), respectively.

##### Adsorption Capacity

Equilibrium adsorption quantity *q*_*e*_ indicates the amount of adsorbate per unit mass of adsorbent when the adsorption equilibrium is reached, the unit is mg/g, and the calculation formula is:

2 $$ \kern0.5em {q}_e=\frac{\left({C}_0-{C}_e\right)V}{m} $$

*V* and *m* represent the volume (mL) of EE2 and adsorbent dosage (mg), respectively.

##### Adsorption Kinetics

Using the quasi-first-order kinetic model and the quasi-second-order kinetic model to linearly fit the experimental data, a simple kinetic analysis of the adsorption of EE2 by copper sulfide can be made. Quasi-first-order kinetic model [22] equation is as follows Eq. (3):

3 $$ \ln {q}_e=\ln \left({q}_e-{q}_t\right)+{K}_1t $$

*q*_*t*_ is the adsorption amount of adsorbent adsorbing EE2 solution at time *t*, the unit is mg/g, and *K*_1_ is the quasi-first-order kinetic adsorption rate constant, the unit is min^−1^. Quasi-second-order kinetic model [23] equation is as follows:

4 $$ \frac{t}{q_t}=\frac{1}{K_2{q}_e^2}+\frac{1}{q_e}t $$

*K*_2_ is the quasi-secondary adsorption rate constant, the unit is g/(mg min).

##### Isothermal Adsorption Model

The isothermal adsorption model is usually used to study the interaction between adsorbent and adsorbate in the adsorption process. There are two common isothermal adsorption models: Langmuir model [24] and Freundlich model [25].

The Langmuir model assumes that the adsorption sites on the surface of the adsorbent are evenly distributed, and the adsorbate forms a single molecular adsorption layer on the surface of the adsorbent. The expression formula is as follows:

5 $$ \frac{1}{q_e}=\frac{1}{q_m{k}_L}\ \frac{1}{C_e}+\frac{1}{q_m} $$

*q*_*m*_ represents the maximum adsorption capacity (or saturated adsorption amount) of the adsorbent to EE2, the unit is mg/g, *k*_*L*_ is the Langmuir constant, which is the ratio of the adsorption rate to the desorption rate, which can reflect the adsorption strength of the adsorbent on the adsorbate, the unit is L/mg.

The Friendlies adsorption model is an empirical formula used to study multilayer adsorption models. Its expression is:

6 $$ \ln {q}_e=\ln {K}_F+\frac{1}{n}\ln {C}_e $$

*K*_*F*_ is the Freundlich constant used to characterize the performance of the adsorbent, and *n* is the reflection of the difficulty of adsorption.

##### Adsorption Thermodynamics

**Fig. 1:**
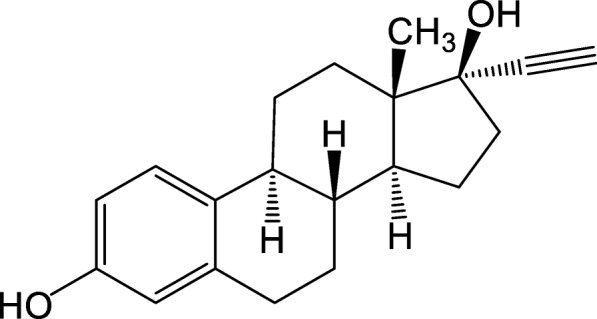
Chemical structure of EE2

The adsorption thermodynamics study was carried out by studying the effect of temperature on the removal of EE2, which provided a deeper understanding of the internal related energy changes during the adsorption process (Fig 1). The thermodynamic description of the adsorption process consists of three parameters: standard Gibbs free energy (Δ*G*^*θ*^), standard thermodynamic enthalpy (Δ*H*^*θ*^), and standard thermodynamic entropy change (Δ*S*^*θ*^) [26]. The relationship between the three is as follows:

7 $$ \Delta {G}^{\theta }=\Delta {H}^{\theta }-T\Delta {S}^{\theta } $$

Further derivation of the above formula can be expressed as:

8 $$ \Delta {G}^{\theta }=- RT\ln {K}_C $$

where *R* is the gas constant, the value is 8.314 J/(mol K); *T* is the adsorption temperature, the unit is *K*; and *K*_*C*_ is the thermodynamic equilibrium constant. The calculation formula is as follows:

9 $$ {K}_C=\frac{C_0-{C}_e}{C_e} $$

In summary, we could get the summary formula:

10 $$ \ln {K}_C=-\frac{\Delta {H}^{\theta }}{RT}+\frac{\Delta {S}^{\theta }}{R} $$

A linear function can be obtained by plotting ln*K*_*C*_ versus − 1/*T*. The values of Δ*H*^*θ*^ and ∆*S*^*θ*^ can be calculated from the slope and intercept of the fitted line.

## Results and Discussions

### Characterization

#### XRD Analysis

**Fig. 2:**
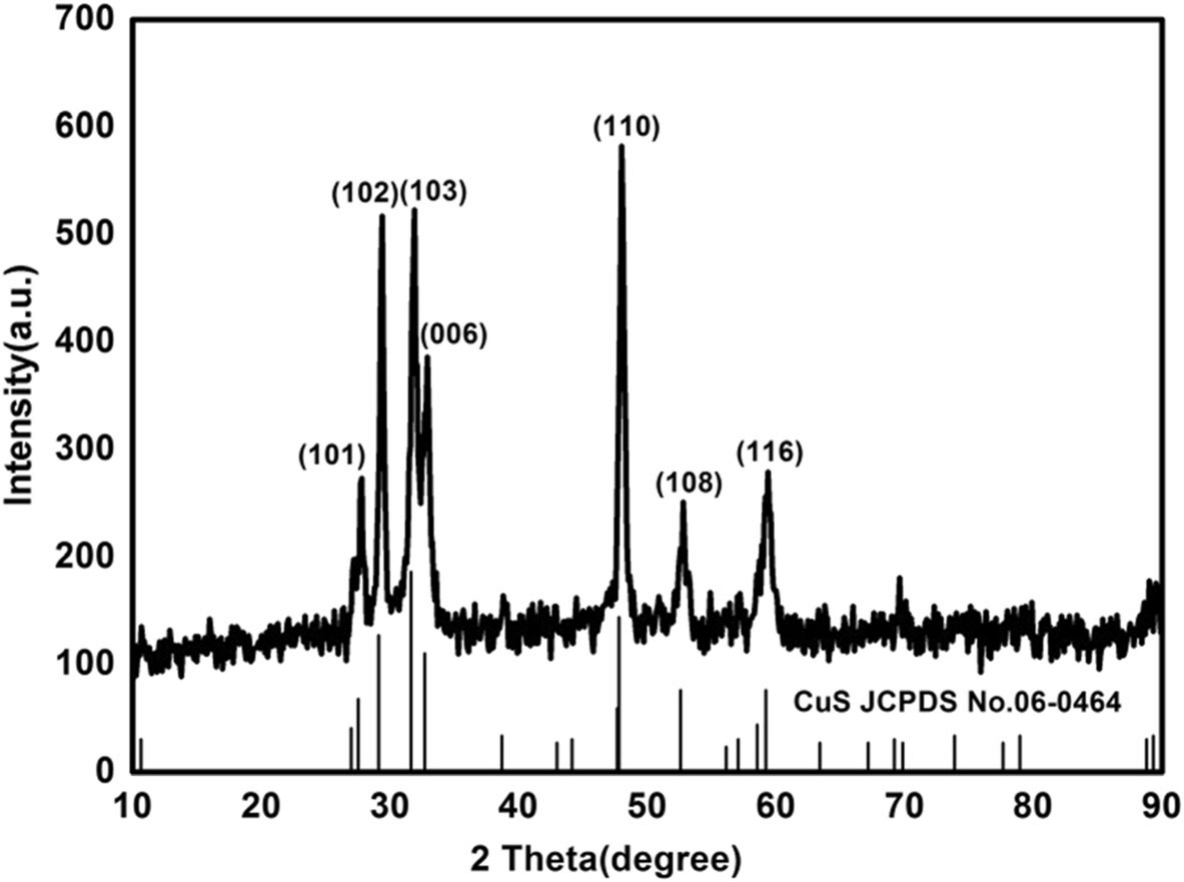
XRD pattern of nano-copper sulfide

The chemical composition and phase structure of materials have been studied using the XRD technique. As shown in Fig. 2, the diffraction peaks of copper sulfide at 2*θ* values of 28, 30, 32, 33, 43, 53, and 59° have been observed, which matched well with (101), (102), (103), (006), (110), (108), and (116) crystal planes of copper sulfide (JCPDS No. 06-0464) [27], respectively. It was proved that pure phase nano-copper sulfide was synthesized in the experiment; no other diffraction peaks were observed, indicating that the material was of high purity.

#### SEM Analysis

**Fig. 3:**
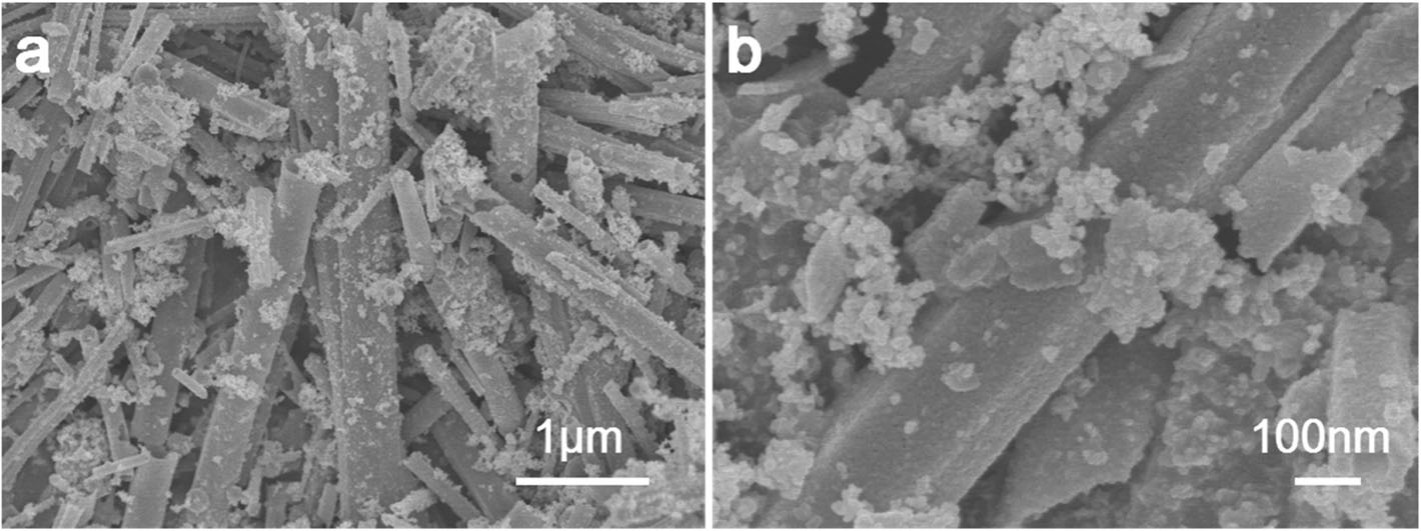
SEM images of nano-copper sulfide

Scanning electron microscopy (SEM) was used to study the morphology of nano-copper sulfide. Figure 3a, b shows the SEM images of copper sulfide at different magnifications. Figure 3a shows that at low magnification, copper sulfide had a hollow tubular structure with a length of 0.4–8.8 μm and a width of 0.1–0.9 μm. Figure 3b shows the microscopic morphology of copper sulfide at a higher magnification; it can be seen from the figure that there are some particle deposits on the tubular structure.

#### TEM Analysis

**Fig. 4:**
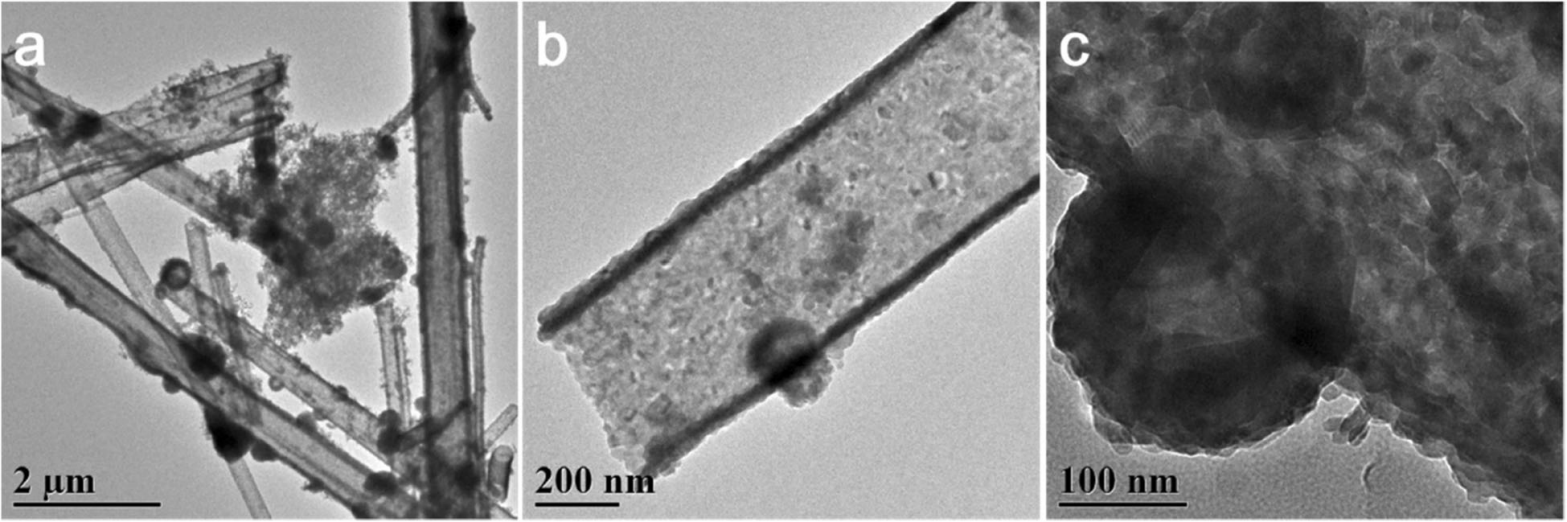
TEM images of copper sulfide

Figure 4 shows the transmission electron micrograph of nano-copper sulfide. It could be seen from Fig. 4a that the tubular copper sulfide was relatively uniform, and the tube diameter was 0.2–0.7 μm. It was apparent from Fig. 4b, c that in addition to the synthetic tubular copper sulfide, particle (spherical) copper sulfide was deposited on the tubular copper sulfide. These results were consistent with the results of SEM. From the SEM and TEM images, it can be known that the synthesized copper sulfide has both tubular and particle (spherical) shapes. Among the two shapes, tubular copper sulfide accounted for the main part, while particle (spherical) copper sulfide was less in quantity, but both shapes of copper sulfide adsorbed EE2.

#### BET Analysis

**Fig. 5:**
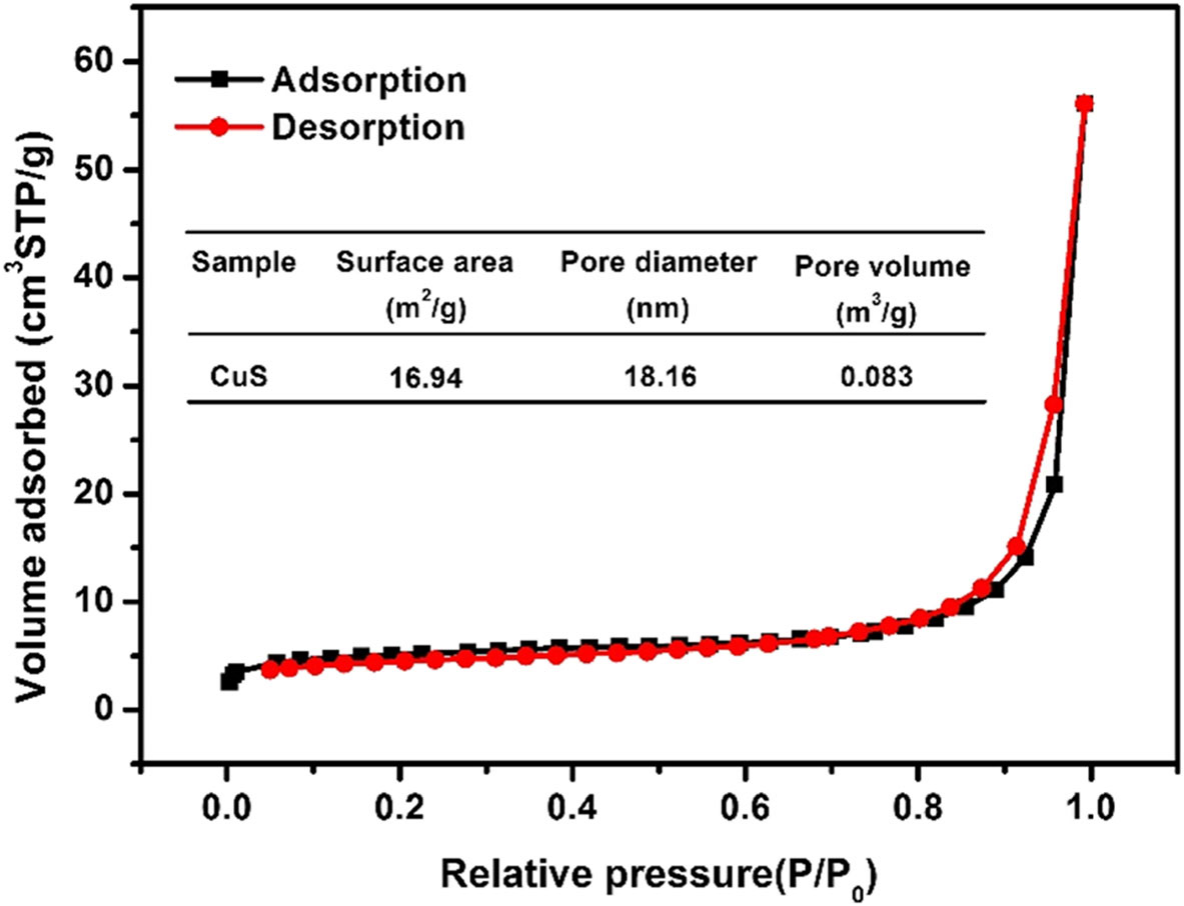
N_2_ adsorption-desorption curve of copper sulfide

The orientation and shape of the N_2_ adsorption-desorption curve can be used to determine the pore structure and pore size distribution of the material. The N_2_ adsorption-desorption curve of the copper sulfide material is shown in Fig. 5. According to the adsorption isotherm classification of Brunauer-Deming-Teller (BDDT) [28], it belonged to the type IV isotherm; the material was mesoporous structure. Generally, the presence of mesoporous structures can provide more surface active sites for the adsorption of active species and reactant molecules, which is beneficial for adsorbing properties. The BET test results showed that the pore size of copper sulfide was 18.16 nm, the specific surface area was 16.94 m^2^/g, and the pore volume was 0.083 m^3^/g. Such a structure and specific surface area were favorable for adsorbing EE2. Combined with SEM and TEM images, it can be known that the synthesized copper sulfide has both tubular and particle (spherical) shapes. Therefore, both shapes of copper sulfide affect the BET measurement.

### Adsorption Experiment

#### Effect of pH on Adsorption

**Fig. 6:**
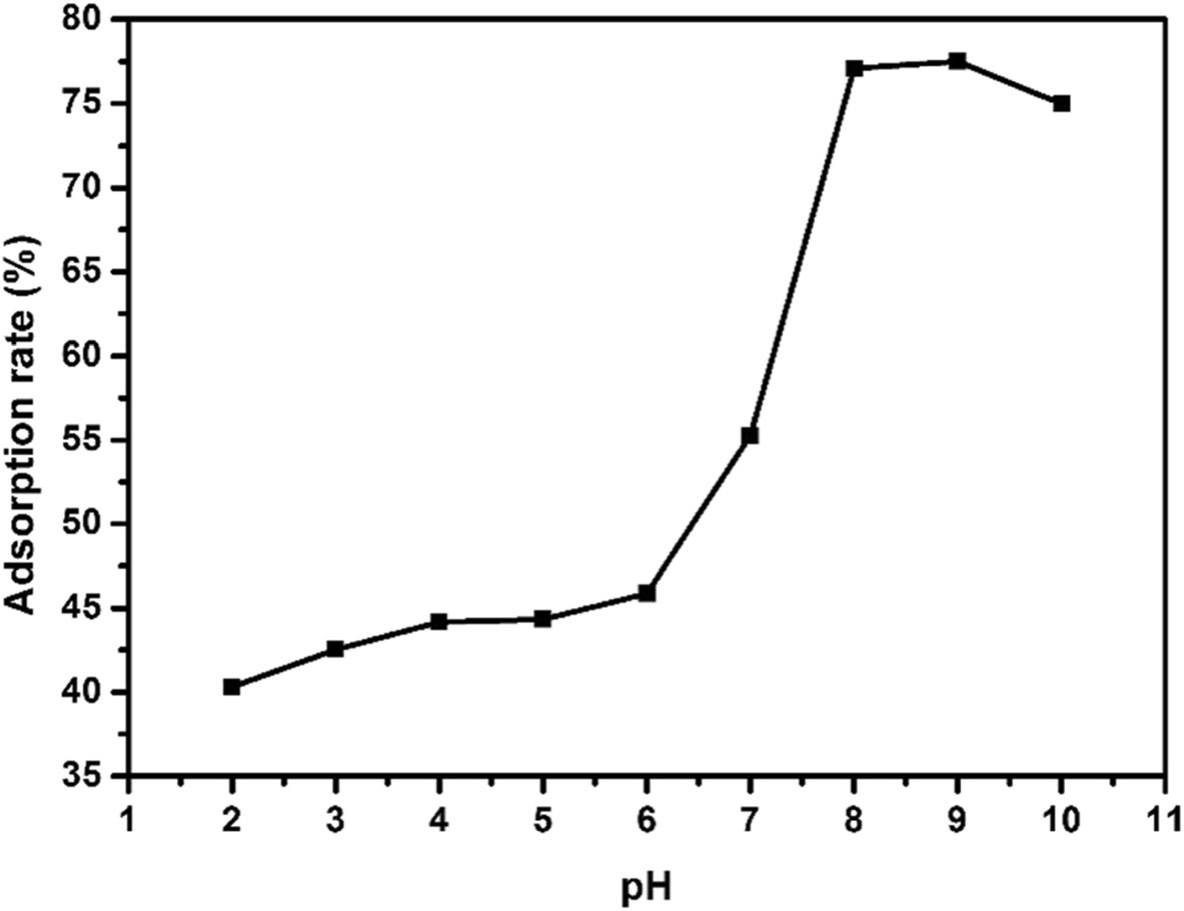
Effect of different pH on adsorption of EE2 by nano-copper sulfide

The influence of pH value of the solution on the adsorption of EE2 was studied by adjusting pH of the solutions with NaOH and HCl. The EE2 adsorption experiments were carried out in the pH range of 2.0–10.0 with adsorbent dosage of 10 mg, initial EE2 concentration of 5 mg/L, shaker temperature of 25 °C, and adsorption time of 3 h. As shown in Fig. 6, the pH was increased from 2 to 6, the adsorption rate of copper sulfide to EE2 did not change much, and the adsorption rate was about 40–45%. Surprisingly, when the pH value was changed to 8, the adsorption rate increased sharply and reached 77.1%.

However, when pH was further increased to 10, the adsorption rate got down to 74.9%. A possible cause of the difference in adsorption rate along the change in pH was that the isoelectric point (IEP) of copper sulfide shifted to the isoelectric point of copper hydroxide (IEP = 9.5) in an alkaline environment [16–18]; at this time, the isoelectric point (IEP) of copper sulfide was relatively large, and its surface was easy to be positive charge [18–20], while there was a phenolic hydroxyl group in the structure of EE2 [21], which can exhibit weak acidity in aqueous solution and negative surface charge, which made strong chemisorption between them. When the pH of the solution was higher than 9.5, the chemical force was reduced and the adsorption rate was correspondingly reduced, which was consistent with the experimental data.

According to the experimental data, pH = 8 was chosen as the optimum pH value for the following experiments.

#### Effect of Adsorbent Dosage on Adsorption

**Fig. 7:**
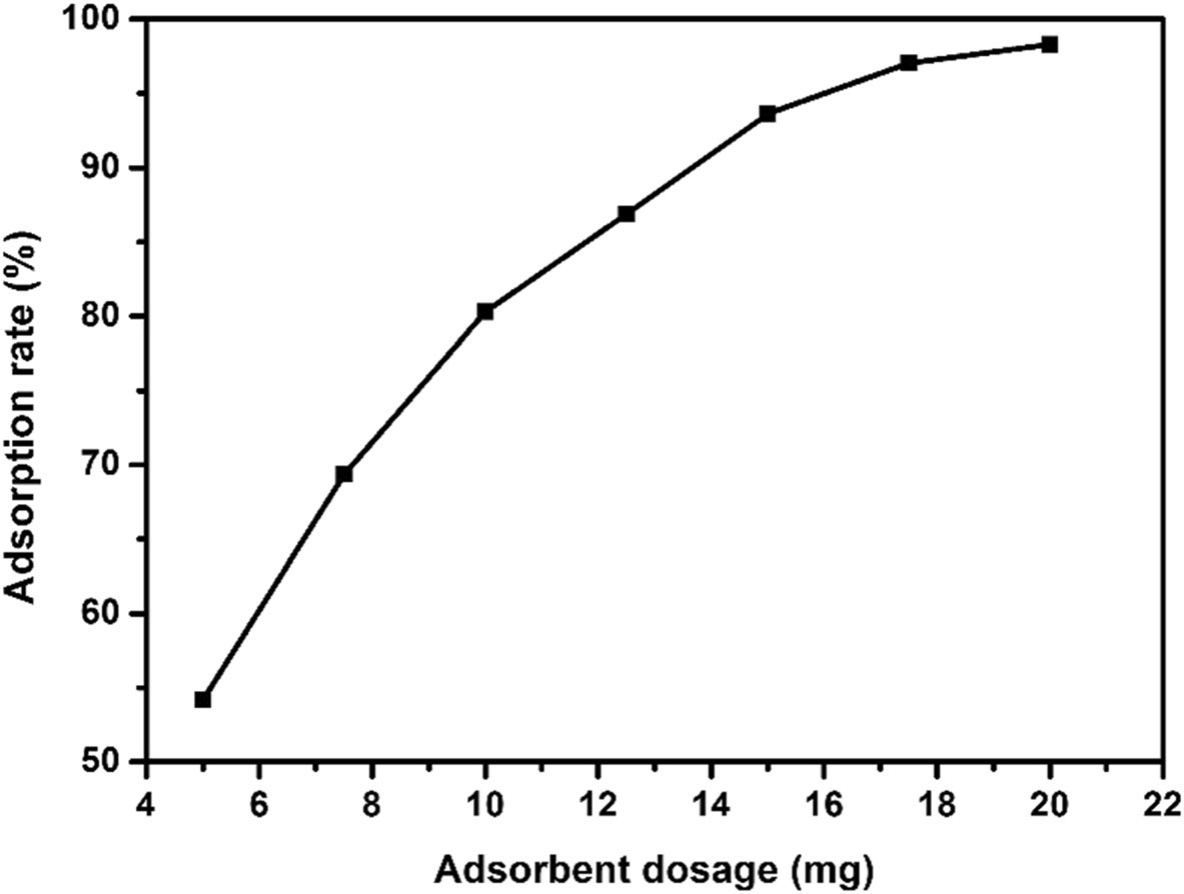
Effect of different copper sulfide adsorbent dosage on adsorption

In order to investigate the effect of different adsorbent dosages on the adsorption of EE2 by copper sulfide, different doses of copper sulfide (5 mg, 7.5 mg, 10 mg, 12.5 mg, 15 mg, 17.5 mg, and 20 mg) were used to adsorb EE2. The EE2 adsorption experiments were carried out at pH = 8 with initial EE2 concentration of 5 mg/L, adsorption temperature of 25 °C, and time of 3 h. As shown in Fig. 7, as the adsorbent dosage increased from 5 to 20 mg, the adsorption rate increased from 54 to 98%. At low doses, the adsorption rates were low due to insufficient adsorption sites, and as the adsorption dose increased, the adsorption sites increased and the adsorption rate increased. When the adsorbed amount was 15 mg, the adsorption rate reached nearly 90%, which was very close to the adsorption rate at adsorbent amount of 20 mg. Considering the economic and environmental issues, adsorbent amount of 15 mg was chosen as the optimized dosage.

#### Effect of Adsorption Time on Adsorption

**Fig. 8:**
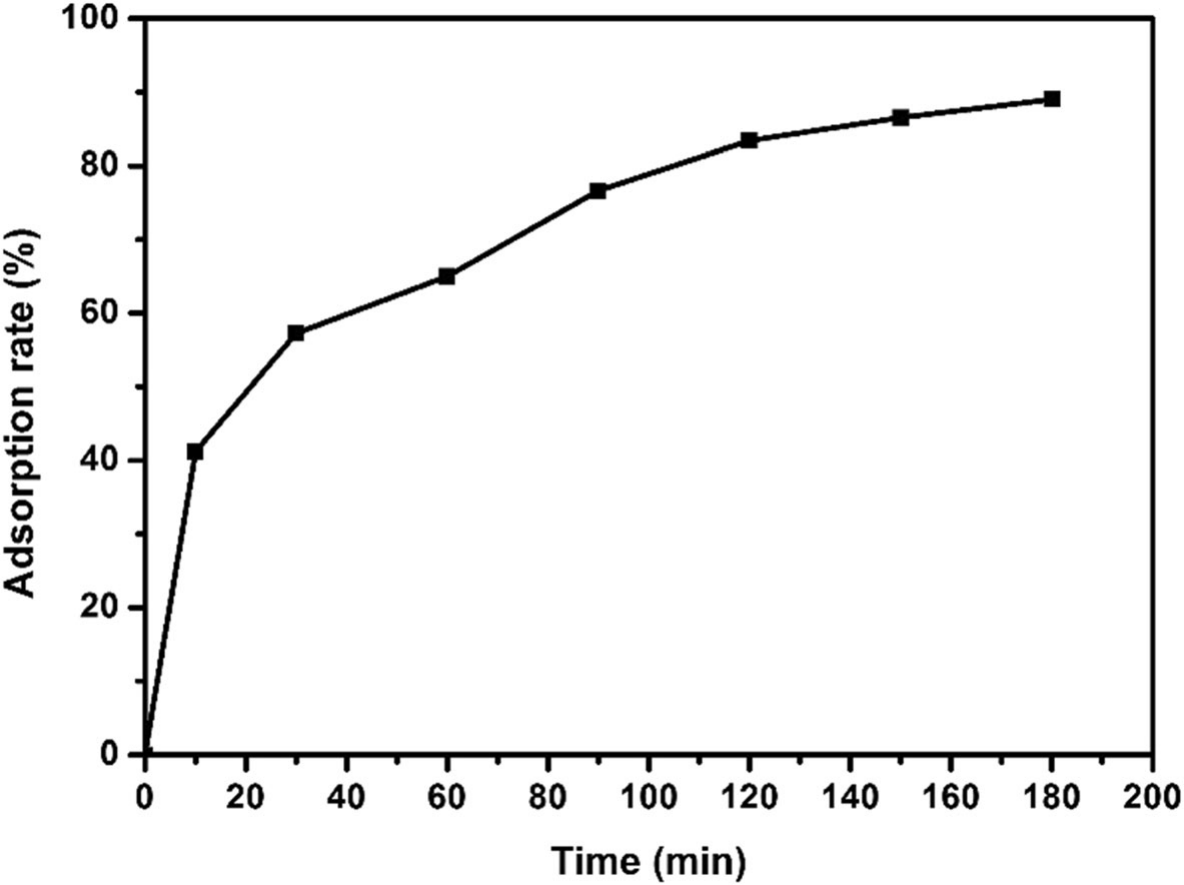
Variation of adsorption rate of EE2 by nano-copper sulfide over time

In order to study the effect of adsorption time on the adsorption rate of EE2 by copper sulfide, adsorption time was set as 0, 10, 30, 60, 90, 120, 150, and 180 min for shaker. The EE2 adsorption experiments were carried out at pH = 8 with adsorption dose of 15 mg, initial EE2 concentration of 5 mg/L, and adsorption temperature of 25 °C. As shown in Fig. 8, adsorption rate of copper sulfide to EE2 reached 89% after adsorbing for 3 h. When the contact time of copper sulfide with EE2 increased, the adsorption removal rate increased.

#### Effect of Temperature on Adsorption

**Fig. 9:**
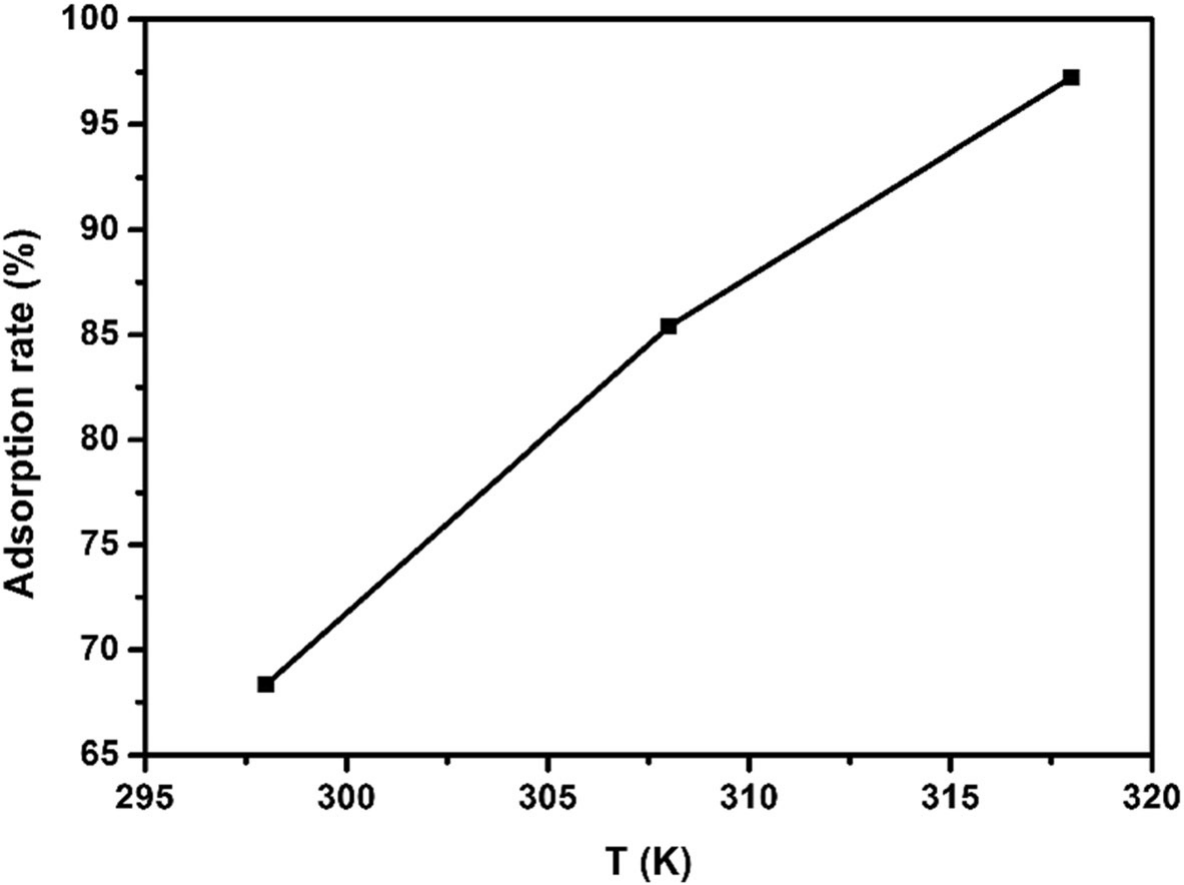
Relationship between different temperatures and adsorption rates

In order to discuss the effect of adsorption temperature on the adsorption of EE2 by copper sulfide, the EE2 adsorption experiments were carried out at 25 °C, 35 °C, and 45 °C. Other experiment conditions were kept the same as follows: pH was 8, adsorption dose was 15 mg, initial EE2 concentration was 5 mg/L, and adsorption time was 3 h. As can be seen in Fig. 9, as the temperature rose from 298 to 318 K, the adsorption rate increased from 68.32 to 97.25%. The results indicated that the reaction was an endothermic reaction.

#### Effect of Initial Concentration of EE2 on Adsorption

**Fig. 10:**
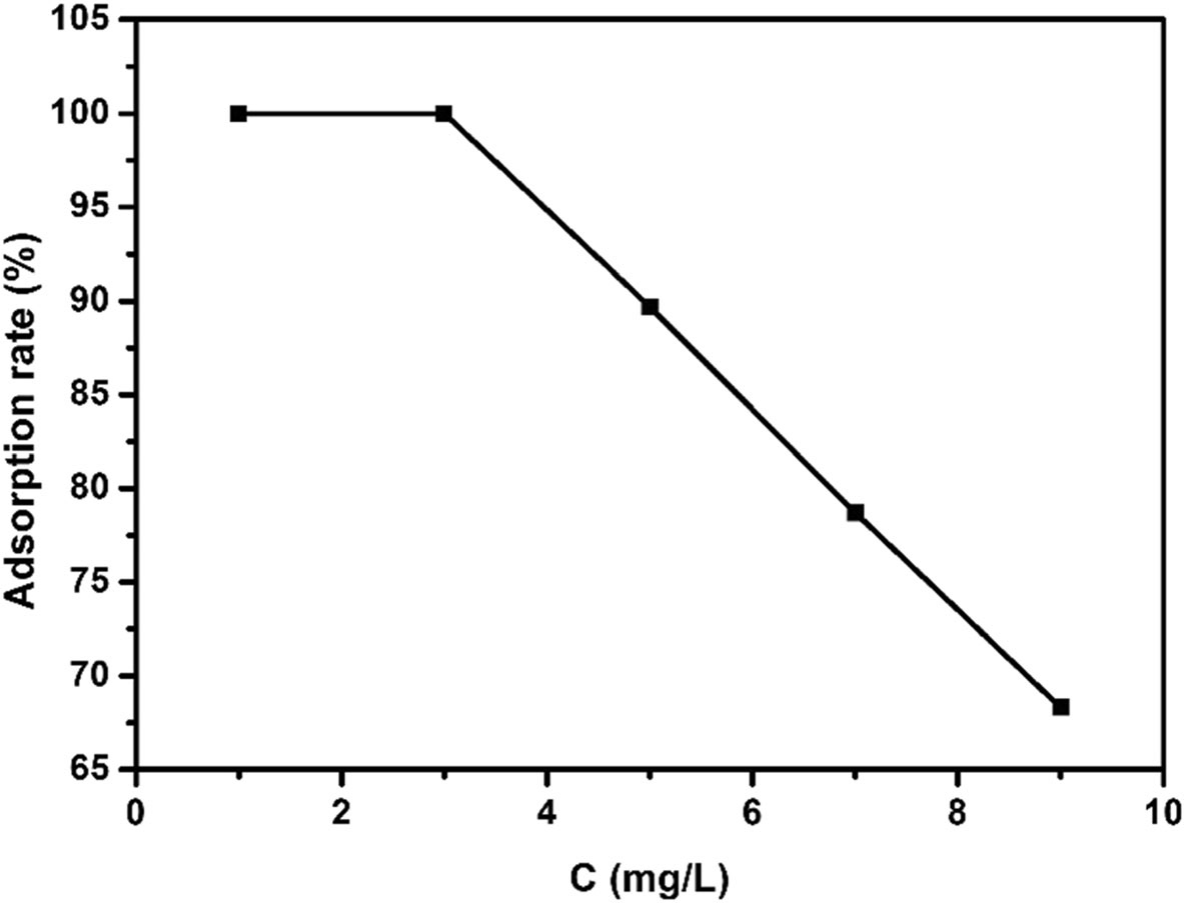
Effect of initial EE2 concentration on adsorption rate

Figure 10 was a graph of different initial concentrations (1, 3, 5, 7, 9 mg/L) of EE2 versus adsorption rates under the conditions of adsorbent dosage of 15 mg, pH = 8, 25 °C, adsorption time of 3 h. It can be seen from the figure that when the initial concentrations of EE2 were 1 mg/L, 3 mg/L, 5 mg/L, 7 mg/L, and 9 mg/L, the adsorption removal rates of copper sulfide to EE2 were 100%, 100%, 89.68%, 78.69. %, and 68.32%, respectively. With the increase of the initial concentration of EE2, the adsorption removal rate of copper sulfide to EE2 gradually decreased. When the initial concentration of EE2 was higher than 3 mg/L, the adsorption rate of EE2 decreased due to the limited amount of catalyst, which cannot provide enough active sites for the high concentration of EE2.

#### Adsorption Stability

**Fig. 11:**
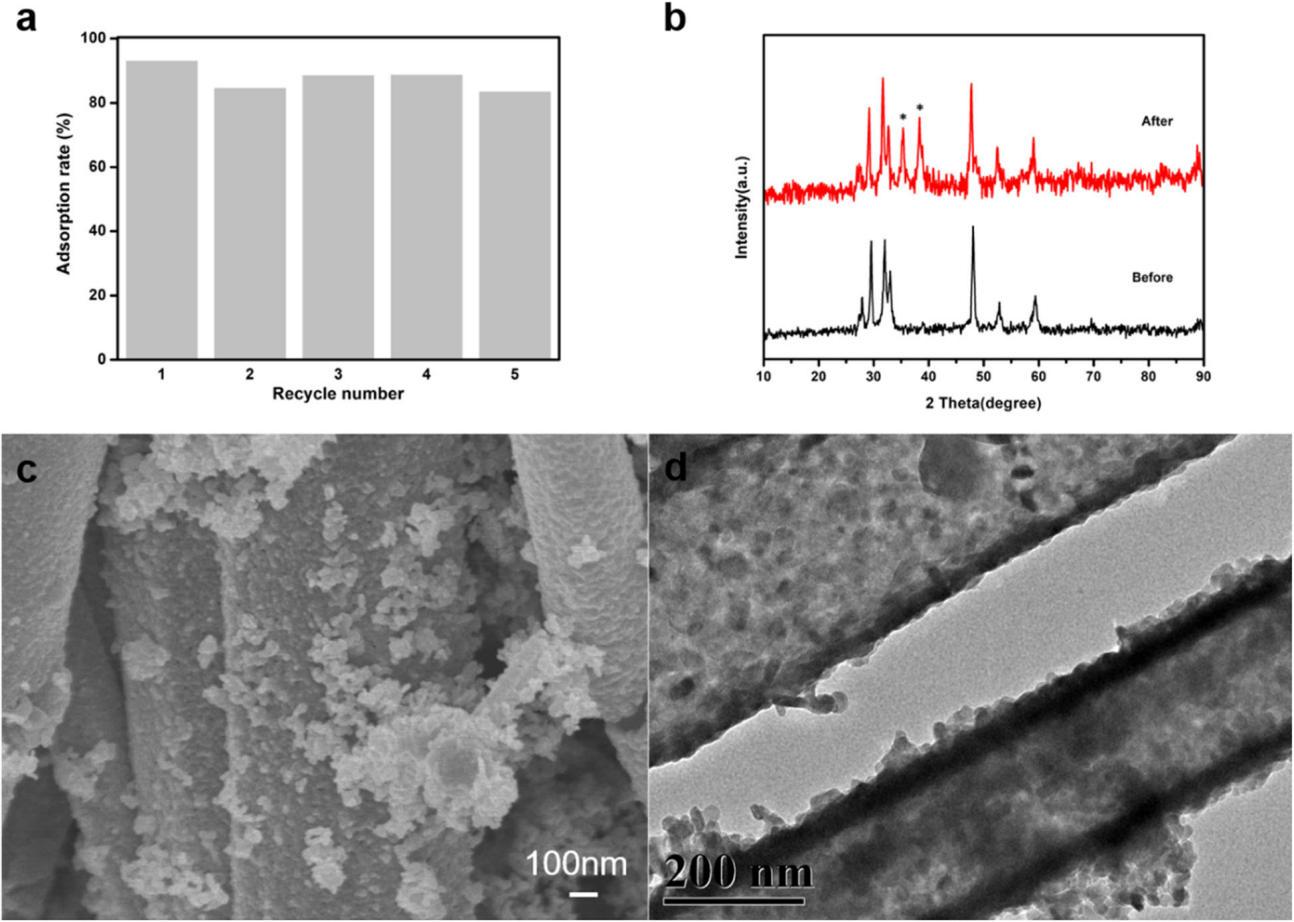
Repeatability experiments of copper sulfide adsorption EE2 (**a**); XRD patterns of CuS, used CuS (**b**); SEM image of used CuS (**c**); and TEM image of used CuS (**d**)

In order to explore the stability of the synthesized nano-copper sulfide, recycling experiments of adsorption of EE2 on copper sulfide were carried out with initial EE2 concentration of 5 mg/L, adsorbent amount of 15 mg, pH of 8, temperature of 25 °C, and adsorption time of 3 h. After each cycle of adsorption, the adsorbent was centrifuged with EE2 aqueous solution, washed alternately with ethanol and water for six times, then dried and reused in the next cycle. It can be seen from Fig. 11a that as the number of repetitions increased, the adsorption rate decreased slightly, but the adsorption rate still exceeded 85%. Figure 11b was the XRD patterns of copper sulfide before and after five cycles. It can be seen from the figure that the phase composition of copper sulfide before and after the cycles slightly changed, and there were two impurity peaks in the marked places in the patterns, which may be the reason for the decrease of adsorption rate after the cycles. It can be seen from the SEM and TEM of copper sulfide in Fig. 11c, d that the morphology of copper sulfide did not change after five cycles and still presented tubular and granular (spherical) shapes.

### Adsorption Mechanism

#### Kinetic Experiment

**Fig. 12:**
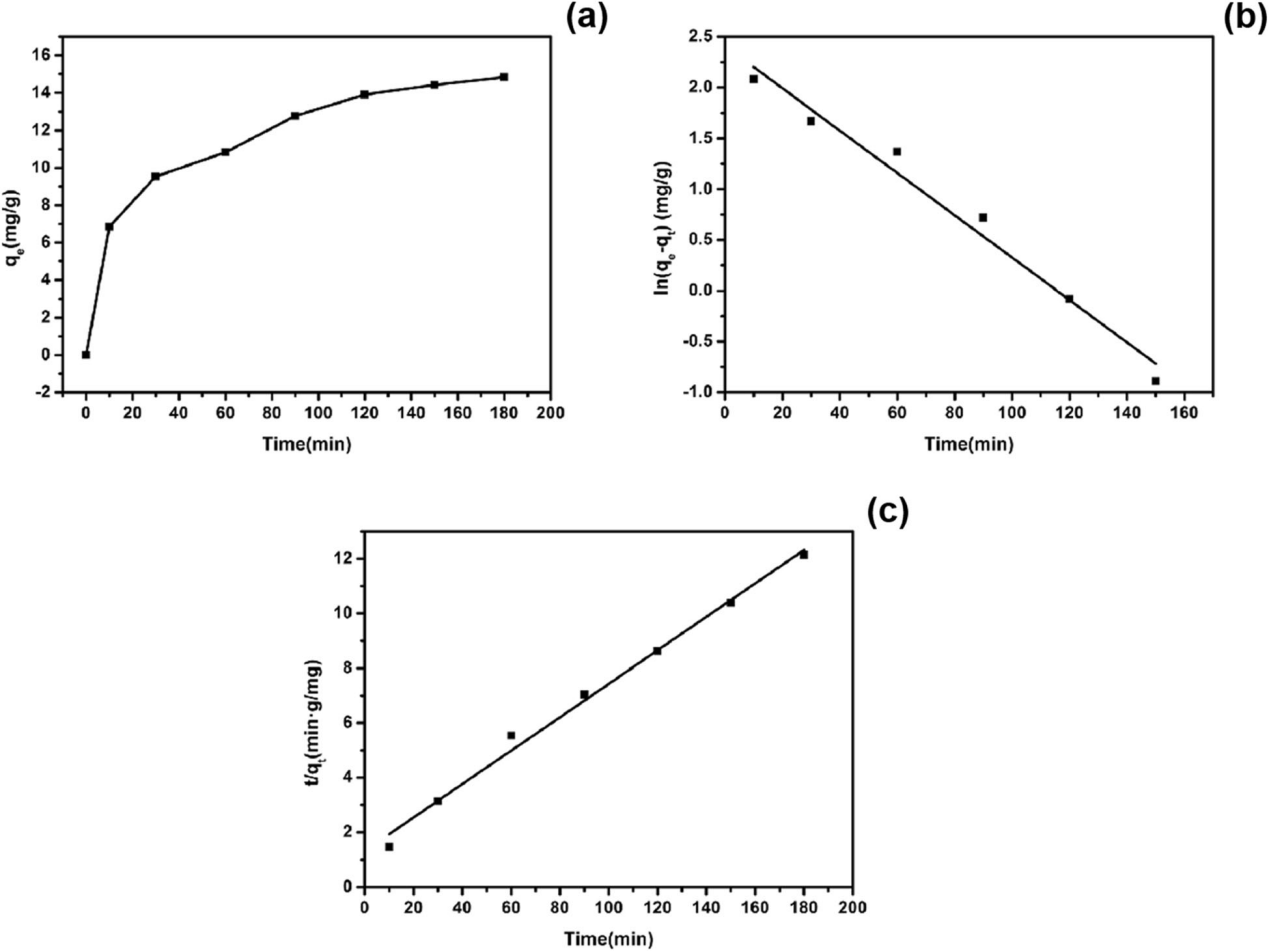
Variation of copper sulfide adsorption with time (**a**), quasi-first-order kinetic model of copper sulfide adsorption EE2 (**b**), and quasi-secondary kinetic model of copper sulfide adsorption EE2 (**c**)

Figure 12a shows the change of the adsorption amount of EE2 adsorbed by copper sulfide with time increasing. It could be seen that the adsorption amount gradually increased with time, but the degree of change gradually reduced. Figure 12b, c shows the first- and second-order kinetic fits of adsorption of EE2 by copper sulfide. Table 1 shows the relevant parameters of the kinetic model. The first-order kinetic equation was obtained by plotting ln (*q*_*e*_−*q*_*t*_) versus *t*, and *K*_1_ was the slope. The second-order kinetic equation was obtained by plotting *t*/*q*_*t*_ versus *t*, and *K*_2_ could be calculated by the intercept. As shown in Table 1, the *R*^2^ of the quasi-first-order kinetics was 0.9784, while the quasi-second-order kinetic model had a *R*^2^ of 0.9916 indicating a better linear relationship. Therefore, the adsorption of EE2 by copper sulfide fits the pseudo-second-order kinetic model better. Moreover, comparing the theoretical equilibrium adsorption amount (*q*_*e*,cal_) calculated by the theoretical equation and the experimentally obtained adsorption amount (*q*_*e*,exp_), their value in the quasi-second-order kinetic model were more close. In summary, the procedure of copper sulfide adsorption EE2 followed the quasi-secondary kinetic model.

**Table 1:** Related parameters of the kinetic model of copper sulfide on EE2

Adsorbent	Quasi-first-order dynamic model				Quasi-secondary dynamics model			
	*K*_1_	*R*^2^	*q*_*e*,cal_ (mg/g)	*q*_*e*,exp_ (mg/g)	*K*_2_	*R*^2^	*q*_*e*,cal_ (mg/g)	*q*_*e*,exp_ (mg/g)
CuS	0.0208	0.9784	11.12	14.83	0.0028	0.9916	16.37	14.83

#### Isothermal Adsorption Experiment

**Fig. 13:**
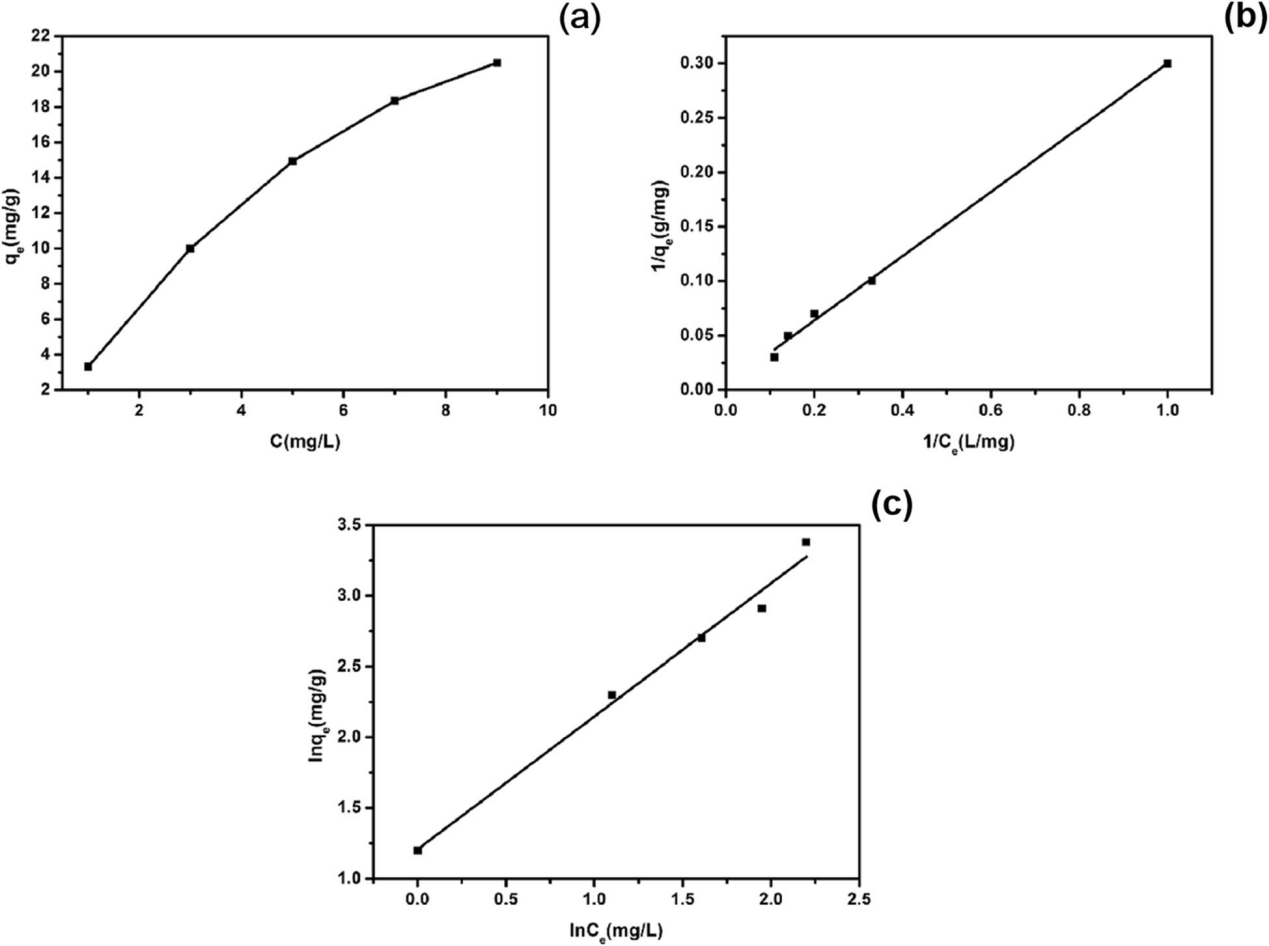
Isotherm curve of 298 K copper sulfide adsorption EE2 (**a**), Langmuir isotherm curve fitting of copper sulfide adsorption EE2 (**b**), and Freundlich isotherm curve fitting of EE2 adsorbed on copper sulfide (**c**)

Figure 13a shows the isotherm adsorption curve of copper sulfide at 298 K. It can be seen from the figure that the higher the concentration of EE2, the larger the adsorption amount. Figure 13b, c shows the Langmuir and Freundlich isothermal fitting curves for the adsorption of EE2 by copper sulfide. Table 2 shows the relevant parameters of Langmuir and Freundlich models. The Langmuir model was plotted a line of 1/*q*_*e*_ versus 1/*C*_*e*_, *q*_*m*_ can be obtained from intercept of the fitted line, and *K*_*L*_ was the slope. The Freundlich model was plotted by the line of ln*C*_e_ versus ln*q*_e_, *K*_F_ was the line intercept, and 1/*n* was the slope. From the relevant parameters in Table 2, it can be seen that the linear correlation coefficient of Langmuir model was better, indicating that the adsorption of EE2 by copper sulfide was more in line with the Langmuir model, and the theoretical maximum adsorption amount *q*_*m*_ of copper sulfide can reach 147.06 mg/g.

**Table 2:** Parameters of EE2 isothermal model for copper sulfide adsorption

Adsorbent	Langmuir isotherm equation			Freundlich isotherm equation		
	*K*_*L*_ (L/mg)	*q*_*m*_ (mg/g)	*R*^2^	*K*_*F*_[(mg/g) (L/mg)^1/*n*^]	1/*n*	*R*^2^
CuS	0.0232	147.06	0.9978	3.3471	0.9402	0.9843

#### Thermodynamic Experiment

**Fig. 14:**
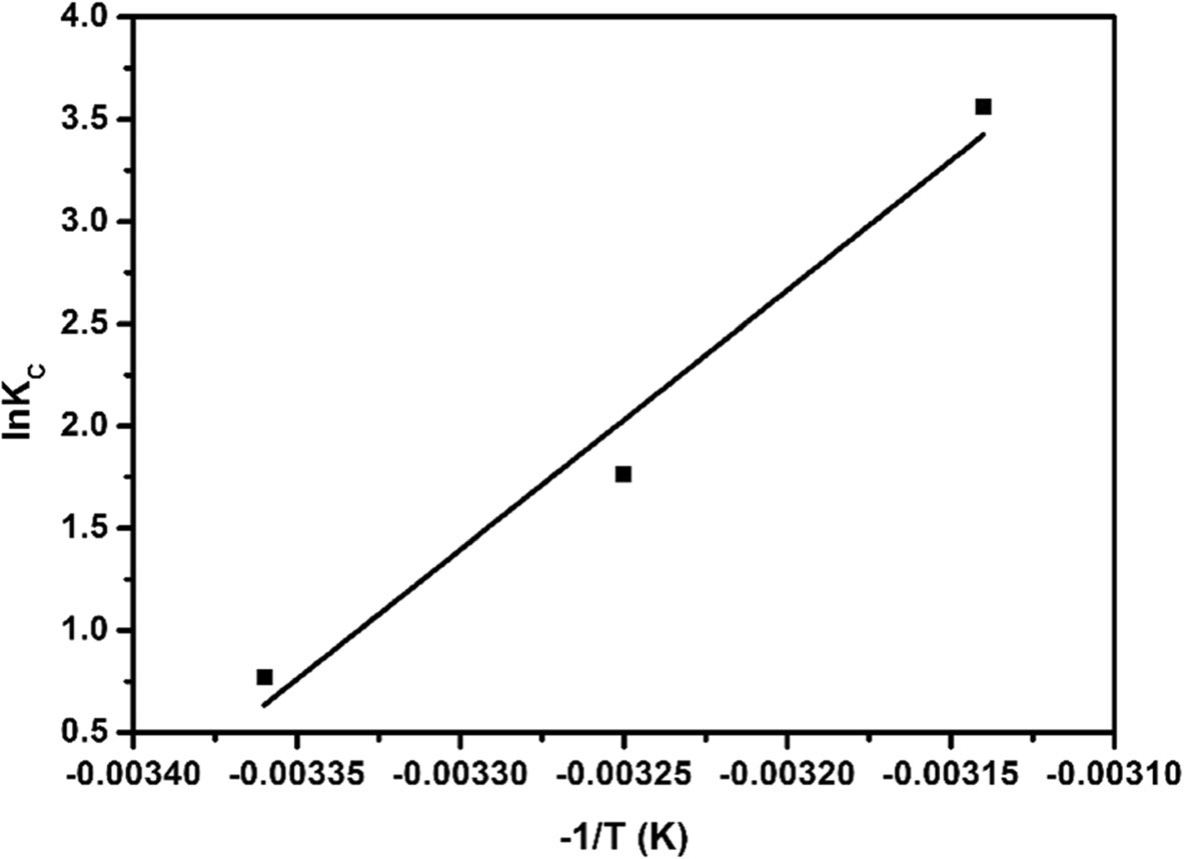
Thermodynamic fit of copper sulfide adsorption EE2

As shown in Fig. 14, in the experiment, a linear fit was performed by ln*K*_*C*_ to − 1/*T*, and the equation ln*K*_*C*_ = 1268.1 (− 1/*T*) + 43.37 was obtained, ∆*H*^*θ*^ was obtained from the slope of the fitted line, and ∆*S*^*θ*^ was obtained by the intercept. Then, the ∆*G*^*θ*^ at 298 K, 308 K, and 318 K were calculated according to formula (7), and the experimental results were shown in Table 3. It can be seen from the table that the Gibbs free energy (∆*G*^*θ*^) of copper sulfide adsorbed EE2 was negative, the thermodynamic enthalpy (∆*H*^*θ*^) was positive, and the entropy (∆*S*^*θ*^) was positive, indicated that the adsorption was a spontaneous endothermic process with increased entropy. According to the literature, the adsorption process of ∆*G*^*θ*^ between − 20 and 0 kJ/mol is physical adsorption, while ∆*G*^*θ*^ between − 400 and − 80 kJ/mol is chemical adsorption process [29]. In Table 3, we can see that the ∆*G*^*θ*^ value calculated according to the thermodynamic experimental data was − 1.84 kJ/mol (298 K), − 5.44 kJ/mol (308 K), − 9.04 kJ/mol (318 K). Therefore, the adsorption of EE2 by copper sulfide belonged to physical adsorption. In the adsorption process, the absolute values of the adsorption heat caused by various adsorption forces were [30, 31]: 4–10 kJ/mol for van der Waals force, 5 kJ/mol for hydrophobic interaction force, 2–40 kJ/mol for hydrogen bond interaction force, and greater than 60 kJ/mol for chemisorption interaction force. The thermodynamic enthalpy (∆*H*^*θ*^ = 105.44 kJ/mol) obtained from the experiment indicated that the adsorption of copper sulfide on EE2 had chemical adsorption characteristics. It could be seen from Table 3 that ∆*S*^*θ*^ > 0, indicating that the adsorption process of copper sulfide on EE2 was a process that increased the chaos of the solution system.

**Table 3:** Thermodynamic fitting parameters of copper sulfide adsorption EE2

Adsorbent	*T* (K)	∆*G*^*θ*^ (kJ/mol)	∆*H*^*θ*^ (kJ/mol)	∆*S*^*θ*^ (kJ/mol)
CuS	298	− 1.84	105.44	0.36
	308	− 5.44		
	318	− 9.04		

## Conclusion

In this paper, the tubular nano-copper sulfide was synthesized by hydrothermal method. The synthesized copper sulfide was used as an adsorbent for 17α-ethynyl estradiol (EE2) and exhibited excellent adsorption properties. At 25 °C, 15 mg of adsorbent was applied for 50 mL of 5 mg/L EE2 solution, in which adsorption equilibrium was achieved after 180 min, and the adsorption rate reached nearly 90%. The adsorption mechanism of copper sulfide material was found to be consistent with the quasi-secondary kinetic model. The isothermal adsorption model was accorded with the Langmuir model, and the maximum theoretical adsorption capacity of copper sulfide was up to 174.06 mg/g. The thermodynamic model study found that the Gibbs free energy ∆*G*^*θ*^ of copper sulfide adsorption EE2 was less than 0, the thermodynamic enthalpy ∆*H*^*θ*^ was greater than 0, and the thermodynamic entropy ∆*S*^*θ*^ was greater than 0, indicating that the whole adsorption process was a spontaneous endothermic process with increased entropy. By studying the values of thermodynamic enthalpy change ∆*H*^*θ*^ and thermodynamic entropy change ∆*G*^*θ*^, it was found that there were chemical adsorption and physical adsorption in the adsorption process. Moreover, the synthesized nano-copper sulfide adsorbent was quite stable under the conditions studied. It is feasible and efficient to absorb EE2 by the nano-copper sulfide adsorbent.

## Data Availability

All data supporting the conclusions of this article are included within the article.

## Abbreviations

BDDT: Brunauer-Deming-Teller
BET: Brunauer-Emmett-Teller measurements
EDCs: Environmental endocrine disruptors
EE2: 17α-Ethynyl estradiol
IEP: Isoelectric point
POPs: Persistent organic pollutants
SEM: Scanning electron microscope
TEM: Transmission electron microscope
UPLC: Ultra-high performance liquid chromatography
XRD: X-ray diffraction
